# Quantitative Measurement of Naïve T Cell Association With Dendritic Cells, FRCs, and Blood Vessels in Lymph Nodes

**DOI:** 10.3389/fimmu.2018.01571

**Published:** 2018-07-26

**Authors:** Humayra Tasnim, G. Matthew Fricke, Janie R. Byrum, Justyna O. Sotiris, Judy L. Cannon, Melanie E. Moses

**Affiliations:** ^1^Moses Biological Computation Laboratory, Department of Computer Science, The University of New Mexico, Albuquerque, NM, United States; ^2^UNM Center for Advanced Research Computing (CARC), The University of New Mexico, Albuquerque, NM, United States; ^3^The Cannon Laboratory, Molecular Genetics & Microbiology, The University of New Mexico, Albuquerque, NM, United States; ^4^Department of Pathology, The University of New Mexico, Albuquerque, NM, United States; ^5^Autophagy, Inflammation, and Metabolism Center of Biomedical Research Excellence, The University of New Mexico, Albuquerque, NM, United States; ^6^Biology Department, The University of New Mexico, Albuquerque, NM, United States; ^7^Santa Fe Institute, Santa Fe, NM, United States

**Keywords:** mutual information, T cells, dendritic cells, FRCs, CCR7, lymph nodes

## Abstract

T cells play a vital role in eliminating pathogenic infections. To activate, naïve T cells search lymph nodes (LNs) for dendritic cells (DCs). Positioning and movement of T cells in LNs is influenced by chemokines including CCL21 as well as multiple cell types and structures in the LNs. Previous studies have suggested that T cell positioning facilitates DC colocalization leading to T:DC interaction. Despite the influence chemical signals, cells, and structures can have on naïve T cell positioning, relatively few studies have used quantitative measures to directly compare T cell interactions with key cell types. Here, we use Pearson correlation coefficient (PCC) and normalized mutual information (NMI) to quantify the extent to which naïve T cells spatially associate with DCs, fibroblastic reticular cells (FRCs), and blood vessels in LNs. We measure spatial associations in physiologically relevant regions. We find that T cells are more spatially associated with FRCs than with their ultimate targets, DCs. We also investigated the role of a key motility chemokine receptor, CCR7, on T cell colocalization with DCs. We find that CCR7 deficiency does not decrease naïve T cell association with DCs, in fact, CCR7^−/−^ T cells show slightly higher DC association compared with wild type T cells. By revealing these associations, we gain insights into factors that drive T cell localization, potentially affecting the timing of productive T:DC interactions and T cell activation.

## 1 Introduction

The adaptive immune response depends on T cell interactions with dendritic cells (DCs) in the paracortex, or T cell zone, of lymph nodes (LNs). The rate at which naïve T cells sample DCs determines how fast the immune system can mount a response to infection (1). The development of imaging methods such as two-photon microscopy (2PM) and histocytometry have enabled direct observation of cell locations in tissues. Many studies showing the relative location of T cells and DCs suggest that they are both positioned in the LN to maximize the likelihood of T:DC interactions (2, 3). Despite advances in the ability to image and observe T cells in LNs, few studies make direct quantitative comparisons of how closely T cells associate with multiple other cells types in LNs.

T cells enter the paracortex of the LN from small post-capillary blood vessels termed high endothelial venules (HEVs). T cells, DCs, and fibroblastic reticular cells (FRCs) occupy this region along with blood vessels (BVs). T cells move among DCs, FRCs, and other T cells to interact with DCs presenting antigen. FRCs are stromal cells that encapsulate a collagen fiber conduit network which allows for transport of lymph fluid carrying soluble antigen and chemokines (4–7). FRCs produce the chemokine CCL21, which has an established role in naïve T cell homing into the paracortex from blood vessels (8, 9). FRCs also provide structural support required for efficient T cell activation (10). Bajenoff et al. showed the FRC network is closely associated with naïve T cells moving within the paracortex, suggesting that FRCs may provide a network on which T cells migrate (11).

There are several hypotheses regarding the role of individual cell types in mediating T:DC interactions. HEVs are the entry points for T cells entering the LN. Girard et al. suggests that DCs gather near HEVs to maximize their contact rate with incoming T cells (12). Others have suggested that DCs may congregate at the intersections of the FRC network, allowing T cells that travel along the edges of the network to encounter DCs at an increased rate (13–16). Spatial interactions between T cells and blood vessels, FRCs, and DCs are important if they change how T cells move through the paracortex and the timing of encounters with antigen-presenting DCs, the key step in T cell activation and the initiation of the adaptive immune response.

In addition to structural and cellular cues, chemical mediators, including chemokines, contribute to T cell motion and T:DC contacts in the LN. For example, the signaling molecule LPA produced by FRCs has been shown to mediate rapid T cell motion in LNs (17). In addition, C–C chemokine receptor type 7 (CCR7), the receptor recognizing CCL21, is important for high speed T cell motility in the LN (18, 19). While CCR7 increases T cell movement speed in LNs, whether CCR7 impacts T:DC contacts has not been investigated.

Understanding the contribution of cellular and structural LN components to T cell localization requires a quantitative metric that allows direct comparisons of spatial associations of multiple cell types. Several other groups have reported spatial relationships between cells and structures using methods such as visual inspection (12, 20) and comparison of turning angles of T cell movements with structures (11, 21). However, none of these directly compare associations between multiple cell types or structures with a consistent quantitative metric.

In this study, we use both the Pearson correlation coefficient [PCC (22, 23)] as well as Mutual Information [MI (24)] to compare the spatial association of multiple cell types and structures. PCC measures the covariance of homologous pixel intensities, and has been often used to determine colocalization, particularly of fluorescent proteins, in multiple biological systems including the study of T cells (25, 26). PCC and MI can be calculated without the need to identify individual cell boundaries which can be difficult for 2PM images.

MI is an application of Shannon entropy (which measures the amount of uncertainty about the value of a random variable in bits) originally defined to understand limitations on signal processing and communication (27). MI quantifies the reduction in uncertainty about one variable when one knows the value of another variable. In analyzing spatial associations, we measure the reduction in uncertainty about the location of one cell type given the location of another cell type. MI has been successfully used in other biomedical image processing applications, particularly in measuring image similarity in X-rays and MRIs for automated image registration (28–31). Furthermore, MI and other information theoretic measures are increasingly recognized as powerful tools for analysis of non-linear complex systems, including complex biological systems such as the immune system (32, 33). In this article, we use MI to quantify the spatial association of T cells with other cell types (e.g., DCs or FRCs). We use MI as a measure of spatial association that is independent of specific types of cells or structures. In addition, MI is theoretically insensitive to coarse graining (34). Thus, MI can measure the amount of spatial dependence of one fluorescent marker on another while minimizing observational bias. MI, unlike distance measures such as nearest-neighbor analysis, is parsimonious, since it does not require extensive image processing to remove photon noise and determine cell boundaries. Instead, MI can operate on the image directly without the introduction of thresholds. In preliminary work we used MI to quantify the association of T cells and DCs and found less correspondence between T cell and DCs than expected (35).

However, MI is not comparable across images with different sizes and amounts of fluorescence. In this study, we use NMI to normalize MI to be between 0 and 1 (36–39), which allows quantitative comparisons of spatial associations between cells fluorescing in one color channel and another cell type fluorescing in a different color channel across experiments. Since PCC and NMI are both pixel-based methods that do not correspond to cell sizes, we create regions within the images that match cellular scales and apply PCC and NMI. Analyzing regions as well as pixels allows these methods to capture associations at biologically relevant scales. Both regional PCC and NMI analyses show T cells associate much less with their ultimate targets, DCs, than with FRCs. Our results also show that CCR7 does not increase T cell association with DCs.

## 2 Materials and Methods

### 2.1 Mice and Reagents

Experiments were performed with C57BL/6 mice (Jackson Laboratories), B6.Ubiquitin-GFP mice (Jackson Laboratories), B6.CCR7^−/−^ mice (Jackson Laboratories) and B6.Cg-Tg(Itgax-Venus)1Mnz/J mice (Jackson Laboratories). Both female and male mice were used between 8 and 20 weeks of age. Breeding, maintenance, and use of animals used in this research conform to the principles outlined by the Institutional Animal Care and Use Committee (IACUC). The IACUC at the University of New Mexico approved the protocol for animal studies (protocol number 16-200497-HSC). Anesthesia via ketamine and xylazine was performed during mouse injections, and euthanasia was administered via isofluorane overdose followed by cervical dislocation. For blood vessel staining, DyLight 594 labeled *Lycopersicon esculentum* (tomato) lectin (Vector Laboratories) was used at a dose of 70 μg per mouse. To isolate naïve T cells, Pan T Cell Isolation Kit II (mouse, Miltenyi Biotec, 130-095-130) was used according to manufacturer’s instructions. To fluorescently label naïve T cells, CellTracker™Orange (5-(and-6)-(((4-chloromethyl)benzoyl)amino)tetramethylrhodamine) (CMTMR) Dye (ThermoFisher Scientific, C2927) was incubated with naïve T cells at a final concentration of 5 μm at 37°C for 30 min before being washed. Labeled naïve T cells were then immediately adoptively transferred into recipient mice.

### 2.2 Mouse Procedures

For all images: 10^7^ naive T cells were adoptively transferred into mice 14–16 h prior to LN harvest for imaging by 2PM. For T:DC images: T cells from naïve wild type (WT) mice were labeled with orange vital dye CMTMR and adoptively transferred into naïve CD11c-yellow fluorescent protein (YFP) mice in which all CD11c^+^ DCs are YFP^+^. For T:BV images: T cells from naïve Ubiquitin-green fluorescent protein (GFP) mice were adoptively transferred into naïve C57Bl/6 recipient mice. DyLight 594-labeled *L. esculentum* (tomato) lectin was injected intravenously into the recipient mice 5 min before harvesting the LNs for imaging. The fluorescent lectin binds to glycoproteins on blood vessel endothelial cells and emits red fluorescence. For T:FRC images: T cells from naïve WT mice were labeled with CMTMR and adoptively transferred into Ubiquitin-GFP recipient mice that were lethally irradiated (10 Gy). The mice were reconstituted with C57Bl/6 bone marrow 4 weeks prior to T cell adoptive transfer. In this chimeric mouse model, the stromal cell populations fluoresce GFP while the hematopoietic cell populations are non-fluorescent.

### 2.3 Two-Photon Microscopy Setup

Two-photon microscopy was performed using either a ZEISS LSM510 META/NLO microscope or Prairie Technologies UltimaMultiphoton microscope from Bruker.

Prairie Technologies UltimaMultiphoton microscope from Bruker: A Ti-Sapphire (Spectra Physics) laser was tuned to either 820 nm for excitation of CMTMR or 850 nm for simultaneous excitation of YFP and CMTMR, GFP and DyLight 594, or GFP and CMTMR excitation. The Prairie system was equipped with Galvo scanning mirrors and an 801 nm long pass dichroic to split excitatory and emitted fluorescence. Emitted fluorescence was separated with a 550 nm long-pass dichroic mirror. Fluorescence below 550 nm was split using a 495 nm dichroic and filtered with 460/60 and 525/50 nm filters before amplification by photo-multiplier tubes. Fluorescence above 550 nm was split with a 640 nm long-pass dichroic mirror before passing through 590/50 and 670/50 nm filters before amplification by GaAsP photo-multiplier tubes. AUMPlanFLN 20× water immersion objective (0.5 numerical aperture) was used. Prairie View 5.4 software (Prairie Technologies) was used to acquire time-lapse z-stacks.

ZEISS LSM510 META/NLO: Chameleon Ti:Sapphire laser tuned to 850 nm (Coherent) was used for excitation of either GFP and CMTMR, YFP and CMTMR, or Dylight 594 and GFP. A 560 nm dichroic mirror and 500–550 and 575–640 nm band pass filters were used for detection of fluorophores. Movies were captured with the ZEN user interface (Zeiss). In both imaging systems, z-stacks with step size of 4 μm were repeatedly imaged over time to obtain movies of 10–45 min in duration. All analyses were performed on 2D image z-stacks captured by 2PM.

### 2.4 Lymph Node Preparation for Live Imaging

After euthanasia, LNs from mice were surgically dissected and transferred to a Chamlide AC-B25 imaging chamber (Live Cell Instruments) with a customized coverslip platform to allow flow beneath the LN. The LN was stabilized with a tissue slice harp (Warner Instruments) and superfused with oxygenated Dulbecco’s Modified Eagle’s Medium (Gibco, 21063-045) and maintained at 37°C. For experiments in which blood vessels were imaged in conjunction with T cells or DCs, with 70 μg DyLight 594-labeled lectin (from *L. esculentum*, Vector Laboratories) was intravenously administered by tail vein injection 5 min before euthanasia.

### 2.5 Calculation of Mutual Information

MI measures how much the value of one variable tells us about the value of another variable. In this study, MI is used to quantify how much the locations and color intensities of DCs, FRCs and blood vessels reveal about the locations and color intensities of T cells. We calculate the MI of color intensities resulting from 2PM imaging of two cell types. Each image is composed of a sequence of 2-color 3D images. In these images one cell type is dyed red and another green. We calculate the MI of the red and green channels from every image to determine the association of the corresponding cell types for that image.

The 2PM images contain red, blue and green channels. For every time step, we extract the red and green channels into two separate 3D images *r* and *g*.

The MI calculation procedure can be summarized in the following 3 steps:

1. We calculate the entropy of color intensities in image *r* and image *g*: H(*r*) and H(*g*). This measures the uncertainty of the color intensity in each image.
2. We calculate the joint entropy H(*r, g*) which measures the uncertainty about the color intensities in corresponding positions in both images.
3. We calculate MI as the sum of the entropies of the individual images H(*r*) and H(*g*) minus the joint entropy of the two images H(*r, g*). This reveals how much uncertainty about the color intensity and location of one cell type (i.e., T cells) is reduced when we know the color intensity and locations of the other cell type.

#### 2.5.1 Entropy

Entropy measures the amount of information in the probability distribution of a random variable (24). It indicates the uncertainty in the outcome of an event. Entropy can be understood by considering a coin toss. The probability of heads is $p(x)=\frac{1}{2}$ and the probability of tails is $p(y)=\frac{1}{2}$. The entropy H is $-\left(\frac{1}{2}\times {\text{log}}_{2}\left(\frac{1}{2}\right)+\frac{1}{2}\times {\text{log}}_{2}\left(\frac{1}{2}\right)\right)$. Since ${\text{log}}_{2}\left(\frac{1}{2}\right)=-1,\text{H=1 bit.}$

The formula for calculating entropy is:

(1) $$\text{H}(r)=-\sum_{r}\text{p(}r{\text{) log}}_{\text{2}}\text{p(}r\text{)},$$

where H(r) is the entropy of variable *r* and *p*(*r*) is the probability of *r* occurring. Here, we use log_2_ so that entropy is measured in bits, the unit of information. The expression is negated because the log_2_ of probabilities (which are always less than or equal to 1) is always negative or 0.

Entropy is maximized for a random event in which the probabilities of all outcomes are equally likely (all *N* possible outcomes have a probability of occurrence of 1/*N*) leading to an entropy of log_2_(*N*) bits. Entropy is minimized for a completely predictable event in which one outcome has a probability of occurrence equal to 1, and all other outcomes have 0 probability of occurrence, leading to an entropy of 0.

We calculate the entropy of color intensities in the red and green images. Each image has 256 possible color intensities for both the red and green images. Thus the maximum H(*r*) and the maximum H(*g*) is log_2_(256) = 8 bits which would occur if each of 256 color intensities were equally likely.

#### 2.5.2 Joint Entropy

We use joint entropy to measure the uncertainty in the outcome of two variables:

(2) $$\text{H}(r,g)=-\sum_{r}\sum_{g}\text{p(}r,g{\text{) log}}_{\text{2}}\text{p(}r,g\text{)},$$

where p(*r, g*) is the joint probability distribution function of *r* and *g*.

The two variables may be unrelated. For example, the joint entropy in the outcome of tossing a fair coin twice is calculated from the probabilities of four possible events [heads, heads], [heads, tails], [tails, heads], and [tails, tails]. The probability of each event is 1/4, resulting in a joint entropy of 2 bits. Since the events are independent, the joint entropy is equal to the sum of the entropies of each individual coin toss.

Alternatively, two variables could be related. In the extreme case, two variables could be completely correlated so that the value of one variable gives perfect information about the value of the other variable. For example, if the second coin toss occurred by picking up the coin and placing it back on the table with the same face up as before, then the probabilities of events [heads, heads] and [tails, tails] are both 1/2, and the probabilities of [heads, tails] and [tails, heads] are both 0. The joint entropy is 1, and equal to either of the individual entropies.

In our analysis of fluorescent images we are interested in the co-occurrence of red and green colors. That is, we wish to know whether knowing the color intensity of green pixels tells us anything about the color intensity of red ones in the same location. We calculate the probabilities of all possible color intensities (0 –255) in all corresponding locations of the red and green images. We define the joint probability p(*r, g*) as the probability of each pair of color intensities (0–255) occurring in the corresponding location in the red and green images. There are 256 × 256 = 65,536 possible combinations of color intensities. We calculate the number of times every intensity combination occurs in corresponding locations in an image. Then, we divide by the total number of locations in the images to turn those occurrences into probabilities. These probabilities are entered in equation (2) to calculate the joint entropy.

The joint entropy is low when color intensities repeatedly co-occur. Note that, joint entropy can be low when either the same color intensities repeatedly overlap, or when different color intensities overlap. For example, if red systematically has lower intensity than green, joint entropy would still be low if a green intensity of, say, 220 was frequently co-located with a red intensity of 180. Joint entropy only depends on the frequency of pairs of values co-occurring in the same locations. Joint entropy is high when there is no association in color intensities between the red and green images. Thus, in Figure 2A where red and green cells are uniformly randomly distributed, there is minimal co-occurrence of the intensities, and therefore all values in the probability table are low and uniformly distributed. By contrast, when red and green cells co-occur with the same intensities in the same locations (Figure 2C), the probabilities on the diagonal are high leading to the minimum possible joint entropy. We observe these scenarios in Figures 2G,I which are the corresponding joint probability tables for Figures 2A,C. For illustration purposes, the 256 color intensity values are binned into 4 color intensities.

**Figure 1:**
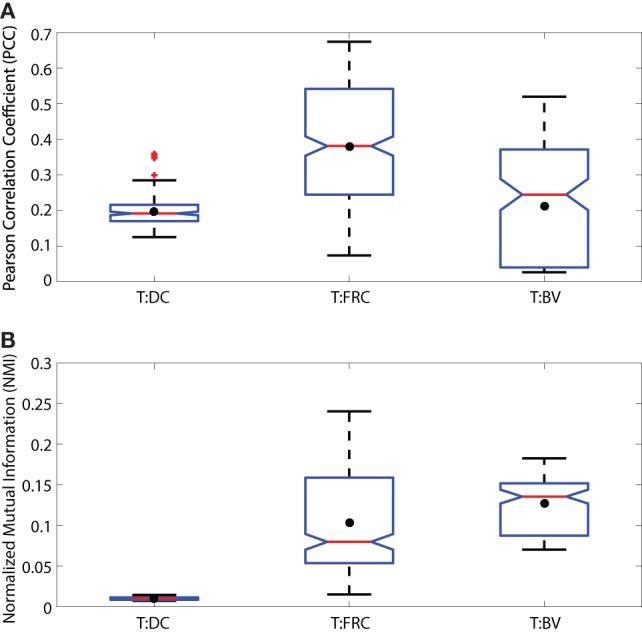
Notched boxplots displaying PCC **(A)** and NMI **(B)** values for T:DC, T:FRC, and T:BV images. Data include 6 T:DC image z-stacks (2 experiments on 2 different days, 2 mice, 4 lymph nodes), 12 T:FRC image z-stacks (3 experiments on 3 different days, 6 lymph nodes), 4 T:BV image z-stacks (2 mice on 2 different days, 3 lymph nodes). Black dots indicate the mean. Median T:DC PCC value = 0.1922, median T:FRCs PCC value = 0.3810, median T:BV PCC value = 0.2447. Mann–Whitney p values for T:DC–T:FRCs < e−4, T:DC–T:BV = 0.0293, and T:FRC-T:BV < e−4. Median T:DC NMI value = 0.0101, median T:FRC NMI value = 0.0798, and median T:BV NMI value = 0.1355. Mann–Whitney p values for T:DC–T:FRC, T:DC–T:BV, and T:FRC-T:BV comparisons < e−4.

**Figure 2:**
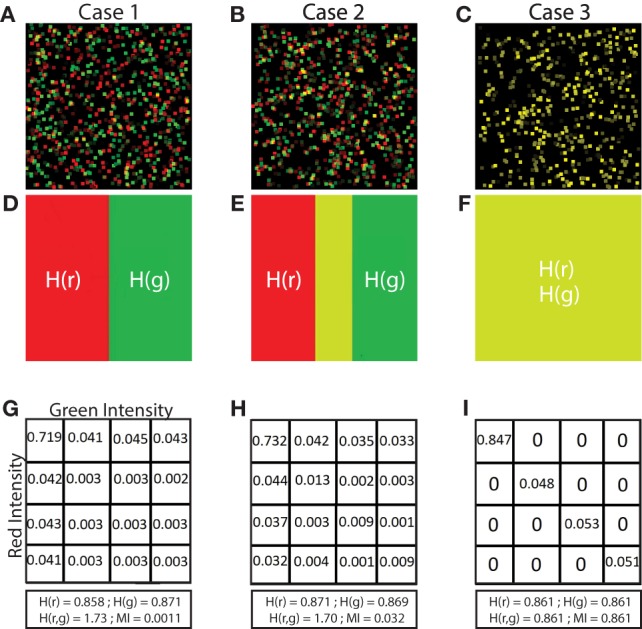
Illustration of low, medium and high MI. Simulated images of 500 red and 500 green cells are shown in panels **(A–C)**. Each cell is a square of 11 × 11 pixels. The location and color intensity of each green cell is chosen from a uniform random distribution. A red cell is paired with each green cell. The red cell has the same color intensity as the green cell, but with a different spatial association in each case. In panel **(A)**, red cells are placed at random locations uncorrelated with green cell placements. In panel **(B)**, the placements of red and green cells are partially correlated. Red cell locations are chosen from a Gaussian distribution centered at the location of the paired green cell, but with a standard deviation (*σ* = 5). In panel **(C)**, the location of red and green cells is identical (*σ* = 0). **(D–F)** Set diagrams indicating the shared information between red and green channels. In panel **(D)**, the two color channels are independent since cell locations are uncorrelated with each other providing minimum MI. In panel **(E)**, the two images are partially correlated which increases the MI, shown by the yellow shaded region. In panel **(F)**, the two images are completely correlated maximizing the MI of the two color channels, resulting in complete intersection of the information in the red and green channels (yellow region). Panels **(G–I)** joint probability tables for images **(A–C)** where 256 color intensities are binned into 4 color intensities for purposes of illustration, resulting in a 4 × 4 probability table. In panel **(G)**, the probability values are low and evenly spread across the table, except for the upper left corner, indicating overlap in the space with no cells (MI = 0.0011 bits). In panel **(H)**, the probability values are higher along the diagonal than in other parts, indicating partial correlation in the placement of red and green cells (MI = 0.0320 bits). In panel **(I)**, there are probability values on the diagonal only and the probabilities off the diagonal are 0 since there is complete correlation in the placement of red and green cells (MI = 0.8610 bits). The calculation of entropy H(*r*) and H(*g*), joint entropy H(*r, g*), and MI are shown for each case.

#### 2.5.3 Mutual Information

MI is calculated from the entropy of each image and the joint entropy of the two images using equation (3).

(3) MI(r,g)=H(r)+H(g)−H(r, g).

Intuitively, this formula calculates MI by subtracting the joint entropy of *r* and *g* from the total entropy in both *r* and *g*, which leaves the overlap in entropy of *r* and *g*.

In Figure 2, we illustrate how MI is calculated from a set of 3 simulated images. The first case (Figure 2A) shows simulated red and green cells placed uniformly in random locations. In most cases, red and green do not overlap as shown in Figure 2D (although by random chance, there is some small co-occurrence of red and green cells that appear yellow). We calculate MI using equation (3). Because there is little or no co-occurrence of red and green pixels in Figure 2A, the joint entropy H(*r, g*) ≈ H(*r*) + H(*g*), so MI ≈ 0.

The second case, in Figure 2B, shows red cells placed within in a Gaussian distributed range of the green cells creating partial co-occurrence of red and green pixels. We can observe this region in Figure 2E (colored in yellow) which is the MI, calculated by summing the entropy of red and green images independently, and then subtracting the joint entropy (equation (2)). The process to calculate the joint entropy of the two images is described in Section 2.5.2 Joint Entropy.

The third case (Figure 2C) is a special case where the red and green pixels are of same intensity residing in the same location. When separated as two images, red and green cells completely overlap, shown in Figure 2F. In this case, information about the location of red cells provides all the information about the location of green cells. Because there is total correspondence between the intensity of red and intensity of green in the same location, the joint entropy H(*r, g*) = H(*r*) = H(*g*), and the MI therefore equals H(*r*) (and also equals H(*g*)).

### 2.6 Normalized Mutual Information

The MI analysis quantifies in bits the amount information shared by images showing the locations of two different cell types. However, the number of bits is influenced by the dimension of images and the numbers and sizes of cells. It does not provide us with a universal scale with which to compare the association of T cells with other cell types. For this, we define and calculate NMI as:

(4) $$\text{NMI}=\frac{\text{MI(}r\text{, }g\text{)}}{\text{min}(\text{H}(r),\text{H}(g))}.$$

We normalize MI by the minimum entropy image. MI depends on both the joint entropy and the internal (marginal) entropies of each color channel. The internal entropies vary across experiments, resulting in MI values that are not directly comparable. We normalize by dividing MI by the minimum of the internal entropies, since it provides an upper bound on MI, for a proof see Ref. (41).

The value of NMI is bounded between 0 and 1, where 0 indicates no occurrence of the red and green cells in the same location as in Figure 2A, and 1 indicates complete colocalization of the red and green cells as shown in Figure 2C. NMI allows us to directly compare spatial association of cells, regardless of the cell types, cell sizes, and image dimensions in our experiments.

**Figure 3:**
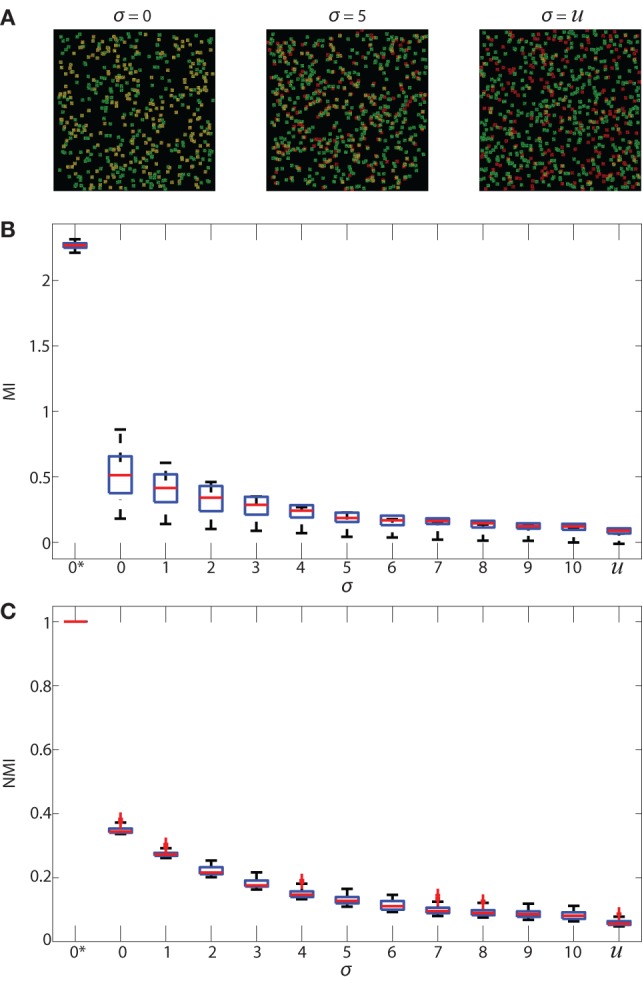
Validation of MI and NMI. Panel **(A)** shows 3 samples of simulated 512 × 512 images that consist of 500 green cells and a number of red cells uniformly distributed between 100 and 500. Each pixel intensity of the red and green cells is randomly assigned, and each cell is a square of 11 × 11 pixels. The red cell locations are chosen from a Gaussian distribution centered at the location of green cells with SD (*σ*) 0 and 5 in the first and second images, and uniformly random in the third image. **(B)** shows boxplots of MI in bits and **(C)** shows boxplots of NMI (unitless) of simulated images where the SD (*σ*) ranges from 0 to 10. 2 additional special cases are shown: 0* and *u*. 0* indicates that red and green color intensities are identical in corresponding locations which maximizes both MI and NMI. *u* indicates that the cells are placed uniformly at random within the image and with uniform random color intensity, resulting in the lowest MI and NMI. Increasing *σ* decreases the spatial association of cells. As spatial association decreases, and both MI and NMI systematically decrease, demonstrating that they are useful metrics that indicate spatial association between cells.

We validated the NMI metric on simulated data generated as 512 × 512 RGB images shown in Figure 3A. Each cell is a square of 11 × 11 pixels with randomly chosen color intensities ranging from 0 to 255. In each image, 500 green cells are placed uniformly at random along with a number of red cells uniformly distributed between 100 and 500. We placed each red cell within a distance determined by a Gaussian distribution from each green cell with SDs (σ) ranging from 0 (generating complete correlation of the red and green pixels) to 10 (generating a low probability of overlap of red and green pixels). We treat the image as a torus to avoid edge effects when placing red cells. We also analyzed images in which both green and red cells are placed uniformly at random (*u*), and therefore with no spatial association and minimum MI.

NMI is designed to normalize for variations in cell numbers. To assess the potential effect of cell numbers on NMI, we simulated images in which we varied the cell numbers from 100 to 500 and calculated NMI for differing cell numbers with complete cell overlap (σ = 0, increasingly spatially separated σ = 1 or σ = 3 or cells placed in a uniform random distribution Figure S2 in Supplementary Material). We also calculated PCC as a comparison. We find that NMI is less sensitive to variations in cell numbers than PCC, particularly in cases in which there is spatial association.

### 2.7 Regionalization of Images

NMI is calculated from the intensity of pixels in corresponding locations. However, cells comprise multiple pixels. A naïve T cell has a diameter of approximately 5–7 μm whereas the approximate length of a pixel is 1.2 μm. Therefore, we created regions in the image and call this process “regionalization.” In regionalization, for each pixel (*p*), we calculated a region around it with a specified length; for example in a 5 × 5 pixel (6 μm × 6 μm) region, *p* is the middle pixel. We replaced the value of *p* with the average color intensity of all cells in its region. We iterated over all pixels, discarding the regions along the image boundaries where complete regions could not be formed. This method produced new images where each pixel has the average intensity of its region. We calculated the MI, NMI, and PCC of these regionalized images. We used region sizes: 5 × 5 pixels (6 μm × 6 μm), 15 × 15 pixels (18 μm × 18 μm), and 25 × 25 pixels (30 μm × 30 μm). We are most interested in region sizes between 5 × 5 (6 μm × 6 μm) and 15 × 15 pixels (18 μm × 18 μm), since these scales are most relevant to our biological data.

**Figure 4:**
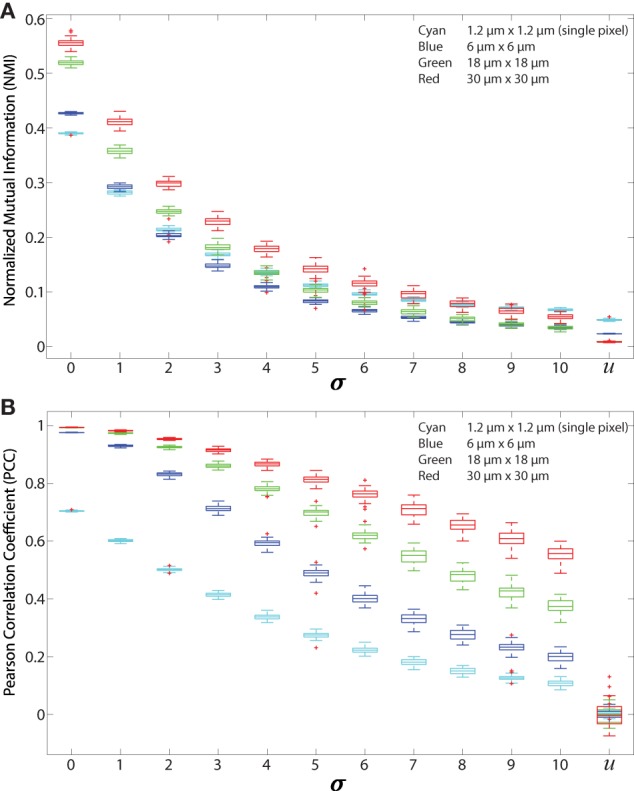
Regionalized PCC and NMI on simulated data. Simulated images are 512 × 512 pixels with 500 red and 500 green 11 × 11 pixel square-shaped cells. The red cell locations are chosen from a Gaussian distribution centered at the location of green cells with SD (*σ*), which ranges from 0 to 10 and *u*. *u* indicates that the cells are placed uniformly at random within the images and with uniform random color intensity. **(A)** NMI calculated on simulated images with regions of sizes 6 μm × 6 μm (blue), 18 μm × 18 μm (green), 30 μm × 30 μm (red), and single pixels (1.2 μm × 1.2 μm, cyan). **(B)** PCC of simulated images using the same regions.

We validated both NMI and PCC for regionalized images. For validation, we used 512 × 512 simulated images that are constructed using the same method mentioned in Section 2.6 Normalized Mutual Information. Analysis is performed on 500 green cells and 500 red cells. These simulated images are then divided into regions using the regionalization method. The size of the regions is consistent with the ones we used for experimental data. Results from NMI and PCC analysis on these images are shown in Figure 4. NMI and PCC decrease with decreasing spatial association, following a trend similar to that in the validation analysis shown in Figure 3, although region size influences PCC more than NMI.

## 3 Results

### 3.1 PCC Shows T Cells Associate More With FRCs Than DCs in LN

To ask whether naïve T cells associate with DCs in the LN, we used PCC, a standard colocalization measure. As a comparison, we also calculated the PCC of T cells and FRCs because it has been suggested that T cells use FRCs as a network for migration through the LN (11). We transferred CMTMR-labeled T cells into CD11c-YFP mice, harvested LNs for 2PM imaging, and calculated PCC of T cells and DCs from multiple images of T cells and DCs. We imaged FRCs as previously described by Bajénoff et al. (11) by irradiating Ubiquitin-GFP animals, reconstituting with whole bone marrow from non-GFP animals for 4–8 weeks, and co-imaged GFP^+^ FRCs with co-transferred CMTMR labeled T cells. We find the PCC of T:DC microscopy images was low (Figure 1A) (median = 0.1916, results given to four significant figures throughout). In fact, the PCC of T cells to DCs was significantly lower than PCC of T cell with FRCs (T:FRC PCC median = 0.3810). In Figure 1, we use interquartile-range notched box plots to visualize the statistical relationships between measurements (42). Non-overlapping notches indicate the measurements were drawn from different distributions at the 95% confidence level. While previous studies have determined association of T cells with FRC and DC subsets separately, we quantitatively compare the effect of FRCs relative to DCs on T cell positioning. These results suggest that FRCs show much higher correlation with naïve T cell locations in the T cell zone of LNs than the presumed intended targets of DCs.

### 3.2 Application and Validation of NMI as a Novel Method to Assess T Cell Association With Cell Types in LN

While PCC provides a quantitative metric to assess the correlation among pixels in images, PCC assumes that these correlations are linear (22, 26, 43, 44). We use NMI (a normalized version of MI) to quantitatively assess spatial relationships between cell types without assuming linearity. The principles of MI are illustrated using simulated images in Figure 2.

We calculated the entropy of fluorescence signals using equation (1) and then calculated the joint entropy using equation (2) (for detail see Methods). We then calculated the MI of the signals using equation (3). To validate our MI calculations, we created simulated images with fields of green and red “cells” in which there is no association (Figure 2A), partial association (Figure 2B), and complete association (Figure 2C) of fluorescent objects with sizes similar to that of cells. The 3 cases can be simplified by observing the images in Figure 2D (no association), Figure 2E (partial association marked as yellow area), and Figure 2F (complete association marked as yellow area). The joint probability tables (simplified examples in 4 × 4 color intensities shown in Figures 2G–I) are used to calculate the joint entropy. If there is no spatial association, the joint probability table shows evenly distributed low values (Figure 2G). Given partial spatial association of cells, the joint probability table shows increased values across the diagonal (Figure 2H). Given completely overlapping signals, the joint probability table shows high values across the diagonal (Figure 2I). Because MI is calculated from fluorescent images in which different images possess different internal entropies, we normalized the MI values to provide a universal scale (between 0 and 1) with which to compare one image to another. We calculated NMI by normalizing MI with the minimum entropy of the two images, thus enabling quantitative comparisons across fields.

In Figure 3A, we show examples of simulated images created for validating NMI (described in Section 2.6 Normalized Mutual Information) in which red cells were placed with SD (σ) of 0 and 5 as well as red cells placed uniformly at random. We expect the MI and NMI values to decrease as the SD increases, as shown in Figure 3B (MI) and Figure 3C (NMI). As expected, MI and NMI are maximum in the special case 0* where the intensity, size and location of the cells are all identical; MI and NMI decrease as the spatial association between the cells decreases. While the MI can be greater than 1 bit (Figure 3B), the NMI metric is normalized to be between 0 and 1 (Figure 3C), demonstrating that NMI can provide comparisons to account for differing levels of fluorescence across multiple fields on a common scale.

As a further validation, we tested whether NMI calculations on our experimental data range between 0 and 1. Figure S1 in Supplementary Material shows that the NMI of an image with itself is 1 (Matched Red:Red and Matched Green:Green). We calculated NMI of two unrelated images from two different experimental fields (Unmatched Red:Green). For example, the red cell image may be taken from a T:DC experiment and the green cell image from a T:FRC experiment. As expected, NMI in these cases is very close to 0 (Figure S1 in Supplementary Material). We then calculated the NMI of T:DC and T:FRC interactions using the same images on which we calculated PCC (Figure 1B). We find that similar to PCC analyses, NMI shows significantly higher association for T:FRC than T:DC (T:FRC NMI median = 0.08; T:DC NMI median = 0.01).

### 3.3 Regional PCC and NMI Analyses

We first calculated both PCC and NMI using pixel-based comparisons (Figure 1). We find that PCC and NMI show a significantly higher association of T cells with FRCs than DCs. However, NMI and PCC pixel-based metrics can be problematic. Intercellular interactions in 2PM images are challenging to quantify by existing colocalization analyses because individual cells occupy discrete physical space, but pixel-based colocalization methods measure the amount of fluorescence signal overlap in individual pixels. In fact, any actual overlap in cell signal as measured by PCC and NMI is likely artifactual in that cells do not physically overlap in space. Also, it is possible that true intercellular contacts would be underestimated due to image resolution and the inability to resolve smaller protrusions such as dendrites of DCs. To account for cell-cell association rather than actual signal overlap based on pixels, we regionalized our images using sliding windows of multiple pixels, the size of which matched approximate sizes of T cells (estimated 5 μm diameter), DCs (estimated 10–15 μm diameter), and FRCs (estimated 5–7 μm diameter). The regionalized image has the same number of pixels as the original, but each pixel contains information drawn from the region surrounding it. Given that each pixel is approximately 1.2 μm in length, we created regions of 5 × 5 pixels (6 μm × 6 μm) and 15 × 15 pixels (18 μm × 18 μm) to account for potential extensions beyond the cell bodies. We also extended analysis to larger region sizes. Fluorescence in regions was determined by taking the average fluorescence of all the pixels within the region (for detail see Section 2.7 Regionalization of Images). We used this method to generate new regionalized images and performed both PCC and NMI to take into account potential interactions of cells without directly overlapping fluorescent signals.

We first tested the “regionalization” effect by performing PCC and NMI on simulated images (as shown in Figures 2A–C and 3A) to determine the effect of cell density, degree of pixel overlap, and regionalization on co-association (Figure 4). We created simulated images that approximate the amount of fluorescence in our experimental images. We varied the distance between the simulated cells to model different amounts of spatial association. We applied our regionalization method to these simulated images and calculated NMI and PCC values. We found that larger regions produce higher NMI and PCC values. Compared with NMI, PCC is less sensitive to changes in spatial association but more sensitive to region size (compare Figures 4A,B). Despite these differences, both NMI and PCC provide a quantitative measure that can be used to detect variation in spatial association among cell types.

### 3.4 Regional Analyses Confirm That T Cells Are More Associated With FRCs Than With DCs

**Figure 5:**
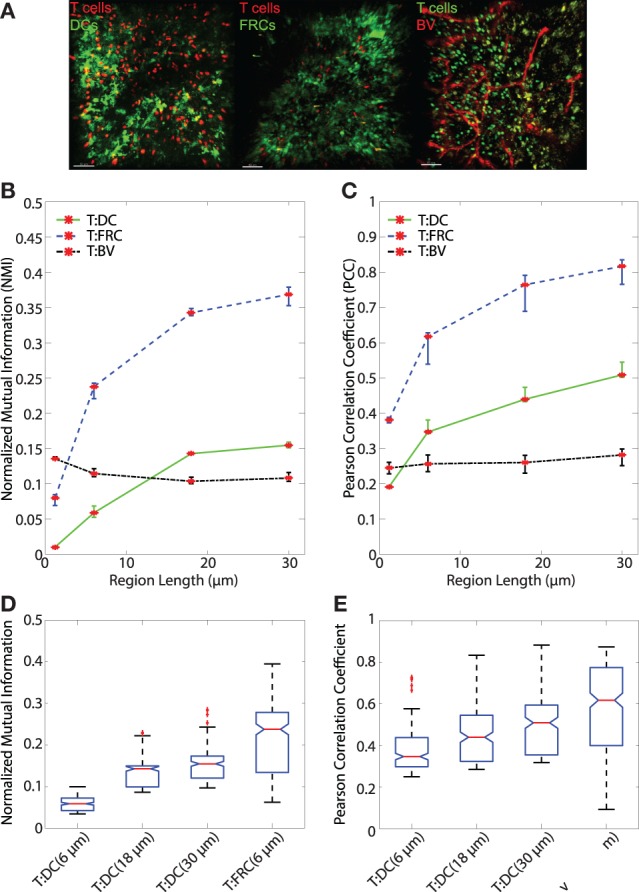
**(A)** Sample images of T:DC (T cells labeled in red and DCs labeled in green), T:FRC (T cells labeled in red and FRCs labeled in green), and T:BV (T cells labeled in green and blood vessel labeled in red). **(B,C)** Line plots representing the NMI **(B)** and PCC **(C)** of T cells and DCs (T:DC, green line), T cells and FRCs (T:FRC, blue dashed line), and T cells and blood vessel (T:BV, black dotted line). NMI and PCC were calculated on pixels (region length = 1.2 μm), or regionalized images of increasing side length (6, 18, and 30 μm). Red stars indicate medians for the corresponding region size, and error bars indicate the 95% confidence interval around the median (40). For NMI, Mann–Whitney p values for T:DC–T:FRC, T:DC–T:BV, and T:FRC-T:BV comparisons < e−4 for all region lengths except T:DC–T:BV (region length = 18 μm) p value = 0.0012. For PCC, Mann–Whitney p values for T:DC–T:FRC, T:DC–T:BV, and T:FRC-T:BV comparisons < e−4 for all region lengths except T:DC–T:BV (region length = 1.2 μm) p value = 0.0293. **(D,E)** Notched box plots comparing the NMI **(D)** and PCC **(E)** of T cells and DCs with T cells and FRCs at physiologically relevant region lengths of (6, 18, and 30 μm) for T:DC associations and 6 μm for T:FRC associations. Note different scales on the y-axis. Both NMI and PCC are greater for the physiologically relevant region sizes for T:FRC than for T:DC (comparing T:DC at 30 μm to T:FRC at 6 μm p = 0.0022; for all other comparisons p < *e–*4). T:DC images were from 6 image z-stacks consisting of 4,089 frames from 2 mice and 4 lymph nodes. T:FRC images were from 12 image z-stacks consisting of 9,468 frames from 3 mice and 6 lymph nodes. T:BV images were from 4 image z-stacks consisting of 4,361 frames from 2 mice and 3 lymph nodes.

After validating both the NMI metric and the regionalization of images, we analyzed regionalized images to quantify spatial association of T cells with DCs and FRCs using both PCC and NMI (for sample images see Figure 5A). Both PCC and NMI show that T cells associate less with DCs than FRCs (Figure 5B for NMI and Figure 5C for PCC). T cells are more associated with FRCs across all region sizes. In pixel-based comparisons (without regionalizing), the T:DC association was very low (Table 1, NMI = 0.0101; PCC = 0.1916) while T:FRC association was significantly higher (NMI = 0.0798; PCC = 0.3810). Both NMI and PCC values for T:DC interactions increased with increasing region sizes, T:FRC association also increased at each region size. Regionalizing PCC into 18 μm × 18 μm region (15 × 15 pixels) resulted in the same trend among the compared cell types as NMI (Figure 5B NMI; T:DC median = 0.1427, T:FRC median = 0.3426; Figure 5C PCC T:DC median = 0.4396, T:FRC median = 0.7646, Table 1). Figures 5D,E compare physiologically relevant regions that approximate cell sizes and account for potential dendritic extensions with larger regions for DCs at 18 and 30 μm than FRCs at 6 μm. Again, T:FRC associations are greater than T:DC associations using both NMI and PCC. Thus, across region sizes, both NMI and PCC analyses show significantly higher T cell association with FRCs compared with DCs. These results suggest that despite the fact that DCs are considered the ultimate targets for T cell search, FRCs a greater determinant of naïve T cell positioning within the LN.

**Table 1:** Median NMI and PCC values among cell types with 95% confidence interval.

Data type	Median NMI	95% Confidence interval	Median PCC	95% Confidence interval
Random control	0.0008	[0.0007, 0.0008]	0.0008	[0.0005, 0.0010]
Same image control	1	[1, 1]	1	[1, 1]
**1.2 μm × 1.2 μm (single pixel)**				
T:DC (WT)	0.0101	[0.0090, 0.0102]	0.1916	[0.1879, 0.1941]
T:DC (CCR7^−/−^)	0.0158	[0.0156, 0.0161]	0.1527	[0.1338, 0.1589]
T:FRC	0.0798	[0.0691, 0.0846]	0.3810	[0.3729, 0.3886]
T:BV	0.1355	[0.1348, 0.1381]	0.2447	[0.2281, 0.2610]
**6 μm × 6 μm**				
T:DC (WT)	0.0588	[0.0524, 0.0685]	0.3467	[0.3427, 0.3808]
T:DC (CCR7^−/−^)	0.0857	[0.0808, 0.0886]	0.4252	[0.3720, 0.4334]
T:FRC	0.2377	[0.2207, 0.2427]	0.6175	[0.5392, 0.6283]
T:BV	0.1144	[0.1101, 0.1214]	0.2565	[0.2342, 0.2815]
**18 μm × 18 μm**				
T:DC (WT)	0.1427	[0.1418, 0.1443]	0.4396	[0.4327, 0.4734]
T:DC (CCR7^−/−^)	0.2633	[0.2576, 0.2679]	0.5866	[0.5794, 0.5957]
T:FRC	0.3426	[0.3384, 0.3487]	0.7646	[0.6893, 0.7913]
T:BV	0.1036	[0.1002, 0.1093]	0.2603	[0.2302,0.2805]
**30 μm × 30 μm**				
T:DC (WT)	0.1547	[0.1509, 0.1589]	0.5089	[0.5020, 0.5448]
T:DC (CCR7^−/−^)	0.3075	[0.2980, 0.3165]	0.6590	[0.6527, 0.6673]
T:FRC	0.3685	[0.3525, 0.3789]	0.8169	[0.7659, 0.8352]
T:BV	0.1080	[0.1034, 0.1159]	0.2816	[0.2514,0.2984]

*Both NMI and PCC values increase with region size except for T:BV*.

In addition to FRCs and DCs, structures such as blood vessels in the LN can be sources of chemokines (5, 45), and T cells may move along vessels in other tissues (21). Several studies suggest DCs are biased to localize near blood vessels and efficiently activate antigen-specific T cells (20, 46). We used NMI and PCC to ask whether vasculature can determine T cell localization in LN. We transferred GFP^+^ T cells for 16 h as previously described, then just prior to imaging, we injected animals with DyLight 594-lectin which binds endothelial cells lining blood vessels. We then imaged T cells in conjunction with vasculature in LNs. With the pixel-based PCC (Figure 1A) and NMI analyses (Figure 1B), T cell association with blood vessels appears higher than T cell association with DCs, and NMI shows higher T cell association with blood vessels than even FRCs. However, with increasing region size, PCC and NMI analyses of T:BV values stayed consistent while T:DC values increased, for example, in the 18 μm length region, NMI of T:DC was 0.1427 and T:BV was 0.1036. The same trend was seen for PCC (T:DC = 0.4396, T:BV = 0.2603). The consistent value of NMI and PCC analyses of T:BV across regions likely reflects the sharp resolution of the blood vessel fluorescence compared with the more blurred extensions of FRCs and DCs. With increasing region size matching cellular scales, T cells show lower association with BVs (Figures 5B,C). These results suggest that T cells likely do not use crawling along vessels as a means to migrate within T cell zones of LNs.

### 3.5 CCR7 Does Not Enhance T:DC Association

**Figure 6:**
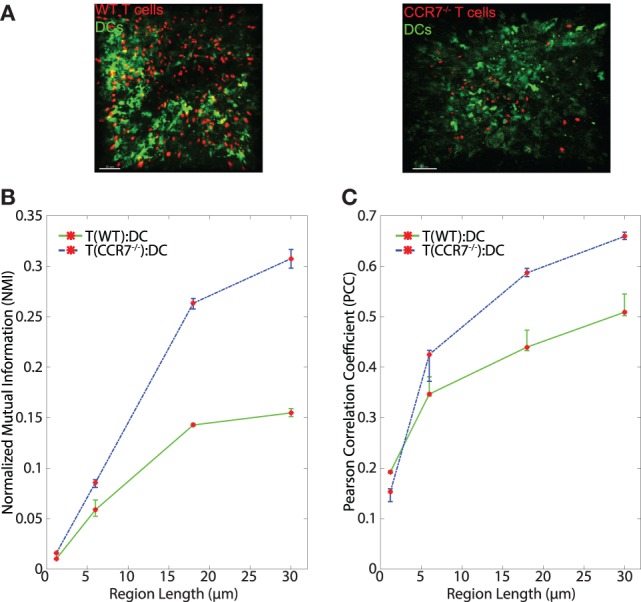
**(A)** Sample images of WT T:DC and CCR7^−/−^ T:DC. T cells are labeled in red and DCs are labeled in green. In WT T:DC, T cells are wild-type naïve T cells and in CCR7^−/−^ T:DC, T cells are from CCR7-deficient animals. **(B,C)** Line plots representing the NMI **(B)** and PCC **(C)** of WT T cells and DCs (T(WT):DC, green line) and CCR7^−/−^ T cells and DCs (T(CCR7^−/−^):DC, blue dashed line). NMI and PCC were calculated on pixels (region length = 1.2 μm), or regionalized images of increasing side length (6, 18, and 30 μm). Red stars indicate medians for the corresponding region size, and error bars indicate the 95% confidence interval around the median (40). For NMI, Mann–Whitney p values for T(WT):DC–T(CCR7^−/−^):DC comparisons < e−4 for all region lengths. For PCC Mann–Whitney p values for T(WT):DC–T(CCR7^−/−^):DC comparisons for region lengths 1.2, 6, 18, and 30 μm: Region length 1.2 μm p < e−4, 6 μm p = 0.9152, 18 μm p = 0.0021, 30 μm p < e−4. WT T:DC images were from 6 image z-stacks consisting of 4,089 frames using 2 mice and 4 lymph nodes. CCR7^−/−^ data are from 12 image z-stacks consisting of 11,294 frames using 4 mice and 8 lymph nodes.

The chemokine CCL21 plays an important role in driving rapid motility of naïve T cells in LNs, and this rapid motility has been suggested to enhance T cell interactions with DCs (3). We tested whether signaling through CCR7 might provide information to T cells to enable closer T:DC associations. To do this, we transferred CMTMR-labeled CCR7^−/−^ T cells into CD11c-YFP mice, harvested LNs for 2PM imaging, and calculated NMI and PCC of CCR7^−/−^ T cells and DCs (Figure 6A). Contrary to our hypothesis, we found that in general, CCR7^−/−^ T cells and DCs showed slightly higher NMI and PCC than WT T:DCs (Figure 6B, NMI WT: 0.0101; CCR7^−/−^: 0.0158 and Table 1). WT T cells showed higher co-association with DCs compared with CCR7^−/−^ T cells in only one case, pixel-based PCC analysis, while with increasing region size and in all NMI analyses, CCR7^−/−^ T cells were slightly increased in DC association over WT T cells (Figures 6B,C; Table 1). Based on both NMI and PCC analyses, these data show that CCR7 does not promote increased T cell localization with DCs. Absence of CCR7 did not increase T:DC association to the level of T:FRC, as NMI and PCC values of T:FRC remained significantly higher than CCR7^−/−^ T:DC association. These results suggest that high speed motility promoted by CCR7 signaling likely functions to promote T cell exploration of the LN paracortex rather than increase T cell localization close to DCs.

## 4 Discussion

In this work, we analyze 2PM z-stacks to quantitatively compare T cell association with different cell types and structures in the naïve lymph node using both PCC and NMI. To account for the limitations of 2PM to resolve cell structures, we create regions that correspond to physiologically relevant cell sizes. Both PCC and NMI across multiple region sizes show that T cells share more spatial association with FRCs than with DCs. Furthermore, CCR7^−/−^ T cells do not associate less with DCs than WT T cells; in fact, our results suggest that CCR7^−/−^ T cells may associate slightly more with DCs than WT T cells.

Many studies have investigated T cell search for DCs in the naïve LN since DCs are the key cell type that is required to present cognate antigen to T cells leading to the initiation of the adaptive immune response (3, 47). Westermann et al. suggest that cell positioning within the LN maximizes the likelihood of T cell interaction with DCs (48). Other studies hypothesize that DCs are situated atop the FRC network to facilitate T cell interactions with DCs as the T cells move along the FRCs (49) and that T cells enter the paracortex from HEVs at specific entry points contiguous with the FRCs network, enabling T cells to be “received” by a greeting line of DCs positioned on top of the FRCs near the HEV entry points (50). Furthermore, different subpopulations of DCs have been shown to localize to specific regions in the LN, suggesting that DC positioning relative to T cells may facilitate T cell activation (51). However, our quantitative analysis using NMI and PCC suggest that T cell association with FRCs does not necessarily lead to similarly high association with DCs. The lack of association between T cells and DCs suggests that T cells have no *a priori* knowledge of DC positions and that DCs are unlikely to attract T cells to DC locations prior to infection. While there is evidence that upon DC activation and infection, chemokines are important to mediate T cell repositioning to DCs (52–54), our data suggests that chemokines CCL19/21 that bind to CCR7 do not play a role in T cell positioning to DCs in the absence of infection. We previously demonstrated that T cells move with a lognormal correlated random walk (55), which aligns with several other studies in the LN (56, 57). Our results suggest that random movement, rather than guided movement, may be the strategy that naive T cells use to interact with DCs prior to infection.

Although T cells and DCs have low NMI and PCC, we find that unexpectedly, lack of CCR7 does not decrease association between T cells and DCs, in fact, CCR7-deficient T cells show slightly increased association with DCs. CCR7 mediates high speed motility in LNs (58). One possible explanation for our finding is that CCR7 deficiency in T cells results in slower T cells that cannot efficiently move away from DCs once they have made contact. Alternatively, CCR7 signaling might be important for T cells to move along FRCs where they receive chemokinetic and survival signals, including both CCL21 and other cytokines such as IL-7 so that in the absence of CCR7, T cells stay closer to DCs, which are not the primary source of CCL21 (59, 60). While it is known that CCR7-deficient T cells are less capable of activation, our quantitative analysis suggests that this may not be due to lack of T:DC contacts but rather may be due to CCR7 effects on overall motility or effects on cosignaling with T cell receptors.

We validated both NMI and PCC on simulated data where we directly manipulated the spatial association of cells and showed that both metrics decrease as spatial association decreases and as region size increases (Figure 4). We designed NMI to normalize for differences in fluorescence between fields, and NMI can quantify non-linear relationships between variables (27) while PCC is based on correlation coefficients (22, 26). In addition, information based measures are theoretically insensitive to coarse graining (34). Our regional NMI analyses in both simulated and experimental images is consistent with this theoretical prediction in that NMI is less sensitive to region size than PCC (Figures 4 and 5). We find that NMI is also less sensitive to variations in cell number than PCC, particularly in cases in which there is already spatial association (Figure S2 in Supplementary Material). Furthermore, NMI based on regions avoids problems associated with pixel-distance measures that arise from 2PM images containing transient single pixel noise (61). Cell-distance measures are also problematic because they require the boundaries of cells, or their centroids, to be well defined. That is usually not the case in 2PM images, especially in the case of DCs and FRCs.

While both NMI and PCC consistently show that T cells are more spatially associated with FRCs than with DCs, we note several caveats in interpreting these results. We considered that T cells may share the highest NMI or PCC with the most numerous cells or structures that occupy the most volume in the paracortex, simply because they cannot move away from the abundant cell type or structure without encountering another cell or structure of the same kind. However, our simulations (Figure 3C) validated that NMI is insensitive to variation in cell number, with fivefold variation in cell number causing much less effect on NMI than changes in spatial association. While the amount of background noise (low-level fluorescence of individual pixels) has some effect on NMI and PCC, that effect does not change the conclusion that NMI and PCC both indicate higher spatial association of T cells with FRCs than with DCs.

Similar to previous studies, our experimental method uses irradiation to image FRCs showing residual GFP^+^ hematopoeitic cells (between 5 and 10%). Thus, it is possible that T:DC can contribute to the T:FRC NMI and PCC. However, because NMI and PCC of T cells with DCs are significantly lower, it is unlikely that the increase in T cell association seen with FRCs is due to residual DC signal. There may also be limitations in the use of two photon imaging as the primary mode of visualizing T cell interactions in the T cell zone as the T cell zone is usually deeper in the LN cortex. Thus, although many publications have used two photon imaging to understand T cell motion in LNs, T cell associations with FRCs and DCs may vary depending on the specific areas that are imaged. In addition, it is possible that staining specific subsets of T cells or DCs may reveal more or less spatial association than we see with total T cells and all CD11c^+^ cells.

In summary, our results show that NMI and PCC both provide quantitative methods to analyze the relationship between two sets of objects, validated in simulations. NMI and PCC show significant differences for different cell populations labeled with two different fluorescent markers, providing quantitative comparisons of fluorescent microscopy images across multiple fields (62). Thus, both NMI and PCC of physiologically relevant regions are useful tools to quantify the relationship between fluorescent cell types. Since MI is a general method for measuring colocalization of fluorescence microscopy images including 2PM signals, the NMI and regional analyses may be broadly applied to any colocalization study of differentially fluorescent objects.

## Software and Data Availability

The code used in this paper is publicly available at: https://github.com/BCLab-UNM/NMIFrontiers2018. The data used is available at: http://digitalrepository.unm.edu/cs_sp/1/.

## Ethics Statement

Breeding, maintenance, and use of animals used in this research conform to the principles outlined by the Institutional Animal Care and Use Committee (IACUC) at the University of New Mexico Health Sciences Center. The IACUC at the University of New Mexico approved the protocol for animal studies (protocol number 16-200497-HSC). Anaesthesia via ketamine and xylazine was performed during mouse injections, and euthanasia was administered via isofluorane overdose followed by cervical dislocation.

## Author Contributions

GMF and MEM conceived of MI as a measure of cell-type interactions. JRB, under JLC, conducted 2PM imaging of *ex vivo* lymph nodes. JRB, HT, JOS, and GMF wrote software to analyze the images. HT wrote a simulation to validate the MI approach. HT, GMF, JRB, MEM, and JLC wrote sections of the paper. HT and JRB created the figures. All authors contributed to manuscript revision and approved the submitted version.

## Conflict of Interest Statement

The authors declare that the research was conducted in the absence of any commercial or financial relationships that could be construed as a potential conflict of interest.

## Supplementary Material

Figure S1 Illustration of the highest and lowest NMI that can be generated from the experimental data. The NMI of an image with itself is the maximum value of 1, shown for an example image of red cells and an example image of green cells. To obtain a minimum value, we calculate NMI between two images, one red and one green from two different fields so that the images are unrelated. We calculated NMI from 5,036 pairs of frames (Unmatched Red:Green). For this unmatched scenario, the NMI is very close to 0 (median is 0.008).

Figure S2 NMI is more robust than PCC to cell count. Simulated images were generated in which numbers of cells in the green and red channels are varied by number and positions varied as indicated. Apparent association of cell types based purely on the increased chance of two cells being near one another as the number of cells goes up is a concern. The normalization factor in NMI is intended to compensate for this artifact. Insensitivity to variation in cell number while preserving sensitivity to the underlying association between cell types distinguishes NMI from PCC. The number of cells in the green channel is kept constant at 500 while the number of cells in the red channel is varied. NMI results are shown in the left column and PCC in the right column. The spatial association between cell types in the model decreases from σ = 0 in the top row to uniform random placement in the bottom row.
