# Supplementary figures and images for: Quantitative Measurement of Naïve T Cell Association With Dendritic Cells, FRCs, and Blood Vessels in Lymph Nodes
Source: Front Immunol. 2018 Jul 26;9:1571. doi: 10.3389/fimmu.2018.01571 (PMC6070610; doi:10.3389/fimmu.2018.01571)

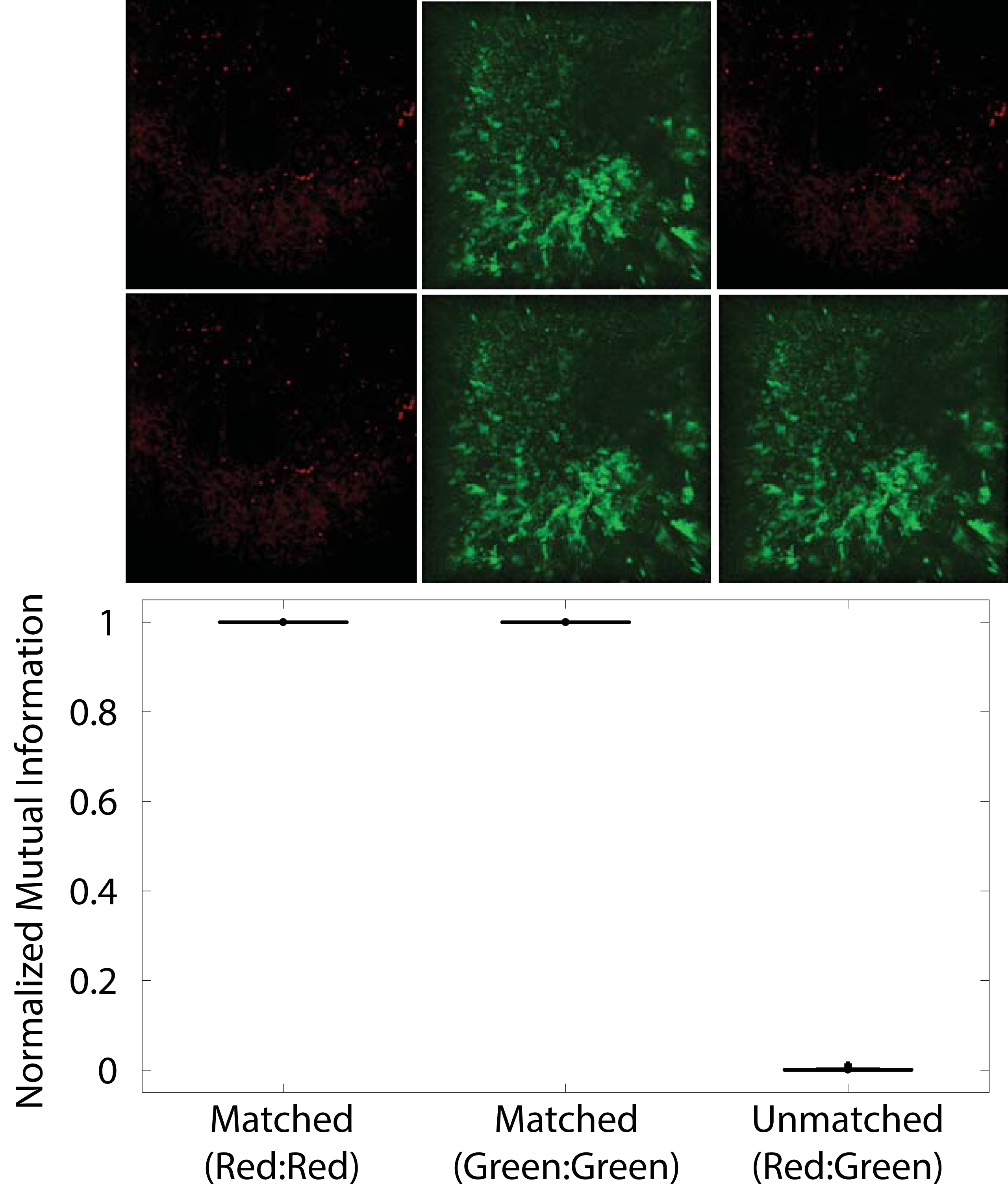

Supplement: Figure S1 — Illustration of the highest and lowest NMI that can be generated from the experimental data. The NMI of an image with itself is the maximum value of 1, shown for an example image of red cells and an example image of green cells. To obtain a minimum value, we calculate NMI between two images, one red and one green from two different fields so that the images are unrelated. We calculated NMI from 5,036 pairs of frames (Unmatched Red:Green). For this unmatched scenario, the NMI is very close to 0 (median is 0.008). [file image_1.tif]

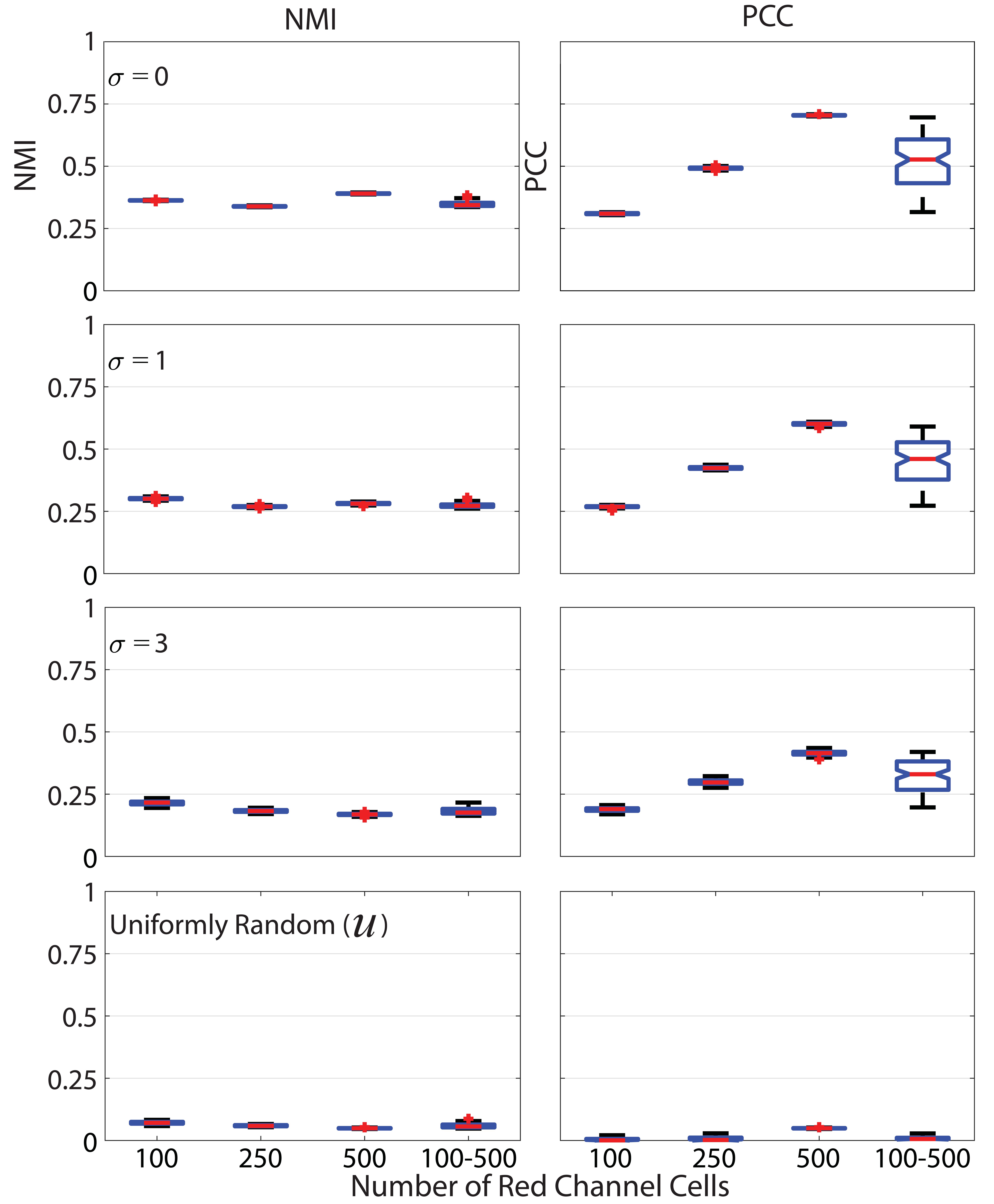

Supplement: Figure S2 — NMI is more robust than PCC to cell count. Simulated images were generated in which numbers of cells in the green and red channels are varied by number and positions varied as indicated. Apparent association of cell types based purely on the increased chance of two cells being near one another as the number of cells goes up is a concern. The normalization factor in NMI is intended to compensate for this artifact. Insensitivity to variation in cell number while preserving sensitivity to the underlying association between cell types distinguishes NMI from PCC. The number of cells in the green channel is kept constant at 500 while the number of cells in the red channel is varied. NMI results are shown in the left column and PCC in the right column. The spatial association between cell types in the model decreases from σ = 0 in the top row to uniform random placement in the bottom row. [file image_2.tif]
